# The nature of prosociality in chimpanzees

**DOI:** 10.1038/ncomms13915

**Published:** 2016-12-20

**Authors:** Claudio Tennie, Keith Jensen, Josep Call

**Affiliations:** 1 School of Psychology, University of Birmingham, Edgbaston, Birmingham B15 2TT, UK; 2 School of Psychological Sciences, University of Manchester, Coupland 1 Building, Coupland Street, Oxford Road, Manchester M13 9PL, UK; 3 School of Psychology & Neuroscience, University of St Andrews, St. Andrews, Fife KY16 9JU, UK; 4 Department of Developmental and Comparative Psychology, Max Planck Institute for Evolutionary Anthropology, Deutscher Platz 6, Leipzig D-04103, Germany

## Abstract

An important debate centres around the nature of prosociality in nonhuman primates. Chimpanzees help other individuals in some experimental settings, yet they do not readily share food. One solution to this paradox is that they are motivated to help others provided there are no competing interests. However, benefits to recipients could arise as by-products of testing. Here we report two studies that separate by-product from intended helping in chimpanzees using a GO/NO-GO paradigm. Actors in one group could help a recipient by releasing a food box, but the same action for another group prevented a recipient from being able to get food. We find no evidence for helping—chimpanzees engaged in the test regardless of the effects on their partners. Illusory prosocial behaviour could arise as a by-product of task design.

## Abstract (web summary)

Chimpanzees appear helpful in some studies yet they do not usually share food, suggesting that they are prosocial when costs are low and goals are clear. Here, Tennie *et al*. show that chimpanzee helping behaviour might be a byproduct of task design and that these apes might not be as prosocial as supposed.

## Graphical abstract

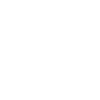

The evolution of behaviour directed at improving the welfare of unrelated individuals, especially when these behaviours are costly for the actor, is an evolutionary puzzle. Although the main conundrum comes from behaviours that decrease an actor's fitness (biological altruism), low-cost behaviours that benefit others are also an anomaly. Typically, non-kin targeted helping is explained by mutualism, reciprocity, policing and reputation which, over the long run, are likely to increase the actor's fitness^1^. Equally puzzling are the motivations underlying prosocial acts. An act is considered prosocial if it is intended to benefit others^2^. This distinguishes genuine acts of prosociality such as helping from self-serving alternatives where benefits to others arise as incidental by-products. To understand the nature and evolutionary origins of prosocial behaviour, recent work has probed social behaviours to determine their possible underlying psychological mechanisms. More specifically, researchers have focused on whether prosocial acts are motivated to foster the welfare of others or instead have a more self-serving nature.

Owing in part to their complex social life, nonhuman primates, and chimpanzees in particular, have been the focus of intense scrutiny with regard to the psychological mechanisms underlying prosocial acts. A number of candidates for prosocial behaviour have been observed in the wild. Chimpanzees risk injury by going on border patrols, come to the aid of each other in conflicts, affiliate with victims of conflicts, groom each other and share food^3^,^4^,^5^,^6^. Even more spectacular examples of potential prosocial behaviour have also been observed, such as adoption of orphans and anecdotal accounts of rescues^7^,^8^,^9^. However, it is difficult to infer intentions from observations alone, particularly anecdotes^10^.

Chimpanzees and other nonhuman primates can receive immediate or delayed benefits from their actions, calling into question whether they have the goal of improving the welfare of others. Grooming can be reciprocated and can be immediately beneficial for the groomer who experiences reduced stress as well as benefits from the parasites they eat^11^,^12^,^13^,^14^. Consoled individuals do not appear to experience reduced stress but consolers benefit by receiving less redirected aggression^15^,^16^. The benefits of border patrols and coalitions, like cooperative hunting, are shared, making these mutualistic interactions. Adoptions typically come long after the orphans lost their mothers^8^. Food transfers tend to be done in response to harassment and begging (manipulation) although active transfers do occur^3^,^17^,^18^.

To address the underlying motivations of apparently prosocial acts, experiments are done in captivity. The experiments fall into two types, sharing and helping. In sharing experiments, subjects typically choose between an outcome that benefits both themselves and a partner or themselves only (mutualistic preference tasks), or between an outcome that benefits a conspecific or no one (altruistic preference tasks). In tasks that involve a food delivery apparatus, chimpanzees rarely transfer food to each other even when there is no cost to doing so^19^,^20^,^21^,^22^,^23^,^24^. A token exchange paradigm involving interactions with human experimenters showed stronger evidence for prosocial preferences^25^, but overall, evidence for food transfers in chimpanzees is weak. Cooperatively breeding monkeys transfer food more often and there has been some evidence from other nonhuman primates, but results are mixed^19^,^26^,^27^,^28^,^29^,^30^,^31^. The strongest evidence for prosocial behaviour comes from experiments of instrumental aid (helping) in chimpanzees. Chimpanzees will hand objects to experimenters who reach for them and will do so in the absence of immediate food rewards, something that capuchin monkeys do not do^32^,^33^,^34^. They also help conspecifics by choosing the correct tool to access a reward^35^, releasing a door to allow conspecifics to pass through^33^ and releasing food and tokens for partners^36^. The implication is that sharing and helping are fundamentally different prosocial behaviours, with the former failing to elicit prosociality in experiments due to competitive motivations for food^37^,^38^,^39^. The ability to infer goals in others—something that chimpanzees, at least, have been shown to do in other situations^40^,^41^—and the motivation to help others achieve those goals, have been suggested to have evolved far earlier than our *Homo* lineage^37^,^38^,^39^.

An alternative interpretation is that there is no difference between the motivation to help and share in chimpanzees and that apparent prosocial behaviour might arise as a by-product of self-regarding preferences. An important feature of helping studies is that subjects might engage with the task due to stimulus enhancement (the socially influenced attraction to environmental features). Stimulus enhancement is an important mechanism for social learning in chimpanzees; observing another individual perform an action on an object can motivate the observer to act on it as well, but without imitating the actions. The same motivation that underlies social learning in chimpanzees—namely attraction to a test due to the actions of other individuals—might also drive apparent prosociality. As well, prior reward histories might also influence the results of the task since in helping studies, subjects first perform the task alone to experience the outcomes themselves; carryover effects could create expectations of rewards^42^.

To address the stimulus enhancement hypothesis, we conducted two experiments on a captive group of chimpanzees that had previously shown evidence of helping behaviours^33^,^36^. Chimpanzees were assigned to one of two groups. In both of these, the subjects (actors) had no access to a food box but the box was accessible to a conspecific (recipient). Actors could release a wooden peg. In the GO group, releasing the peg unlocked the food box, allowing food to be shaken out. Doing so in the NO-GO group had the opposite effect—it locked the food box, preventing the recipient from getting food. If chimpanzees are helpful, actors in the GO group were expected to release the peg more often than the NO-GO group in both experiments. Alternatively, if chimpanzees are spiteful—or competitively motivated by food—they would show the reverse pattern^43^,^44^. A lack of a difference between the behaviour of chimpanzees in the GO and NO-GO groups would lend support to the stimulus enhancement hypothesis and thus call into question the nature of prosocial behaviour in chimpanzees. Actors were first tested without prior experience with the food box (experiment 1). This is an important innovation; all prior apparatus-based experiments on nonhuman primate prosociality have trained subjects on the contingencies nonsocially before introducing a test partner^20^,^21^,^22^,^23^,^24^,^26^,^27^,^28^,^29^,^30^,^31^,^34^,^35^,^36^. Subjects in these studies are therefore reinforced for some choices more than others, and these learning effects may carry over into the tests. Experiment 1 allowed us to determine what the chimpanzees learned solely through observing the effects of their actions on conspecifics. Later, in experiment 2, we provided them with training as in all other tests of prosociality by giving them access to the food box prior to testing; in this way, they learned the consequences of their actions through personal experience.

The key finding is that there was no difference between the two groups of chimpanzees. They were just as likely to release a peg to prevent access to food (NO-GO group) as they were to provide access (GO group). Response rates in both groups declined over time in experiment 1. After personal experience with the apparatuses, chimpanzees only released the peg when doing so resulted in them getting food for themselves. Stimulus enhancement can fully account for apparent prosociality in chimpanzees; prior evidence for prosocial behaviour may have been by-products of experimental designs, producing an illusion of helping in our closest living relatives.

## Results

### Experiment 1

**Figure 1:**
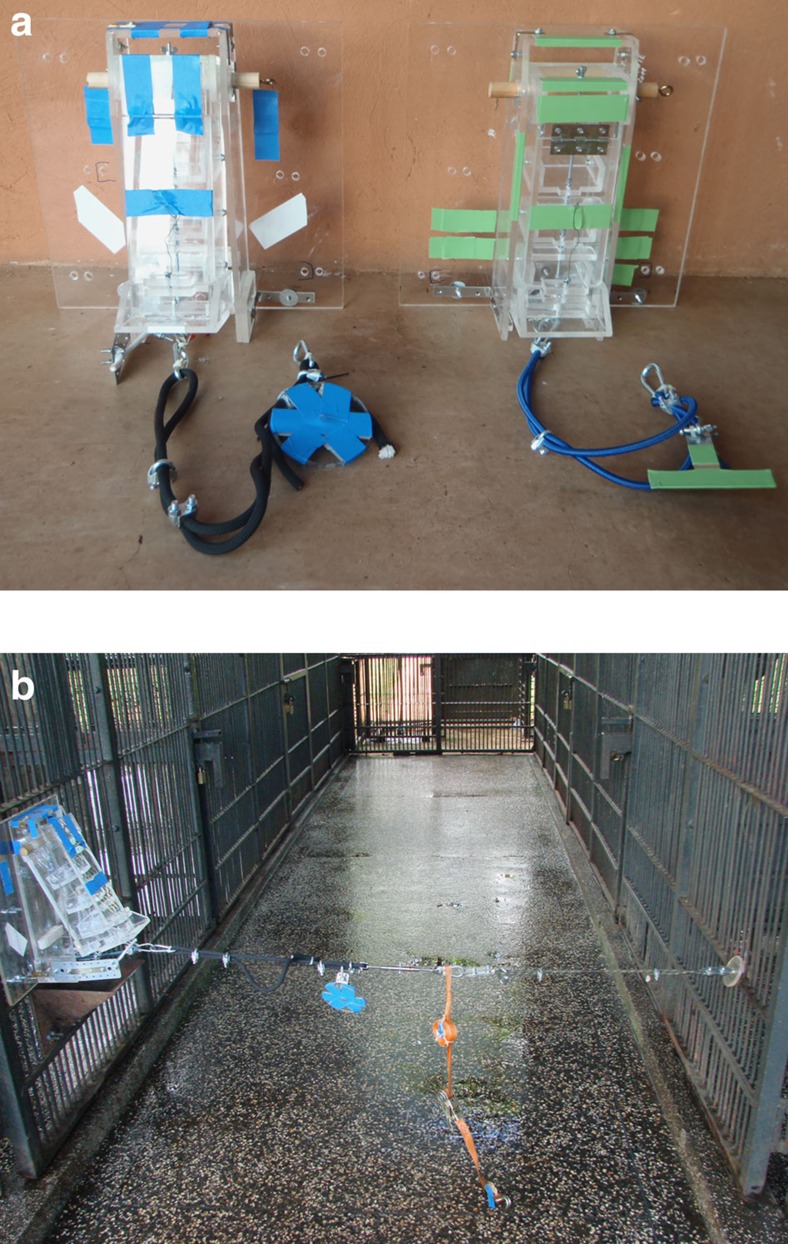
Experimental apparatuses and setup. (**a**) Chimpanzees in the GO group (blue food box on left) could release a peg, allowing food to be shaken out, whereas those in the NO-GO group (green food box on right) would prevent food from being shaken out. (**b**) In the test conditions, recipients would sit in front of the food box (left of image) and actors would face them across a keeper's corridor (right); the peg was attached to the mesh of the actor's room (right).

In experiment 1, six chimpanzees (3 males, 3 females—mean age 13 years) were randomly assigned to be actors in the GO group and seven (4 males, 3 females—mean age 13 years) to the NO-GO group (see Supplementary Table 1 for further details). To minimize the effects of personal relationships, three male chimpanzees of a similar age range were chosen to be recipients. None of the chimpanzees were genetically related. Recipients were individually given experience getting food out of the food boxes before being paired with the actors. In addition to the peg connected to the food box (Fig. 1), a distractor rope and peg were placed in the actor's room (peg room); actors were also given a towel soaked in fruit juice. The purpose of the distractors, which were identical to those used in previous helping studies^33^,^36^, was to lower the rate of peg releasing below ceiling levels. Each pair was tested across four sessions of four trials each (see Methods for details).

**Figure 2:**
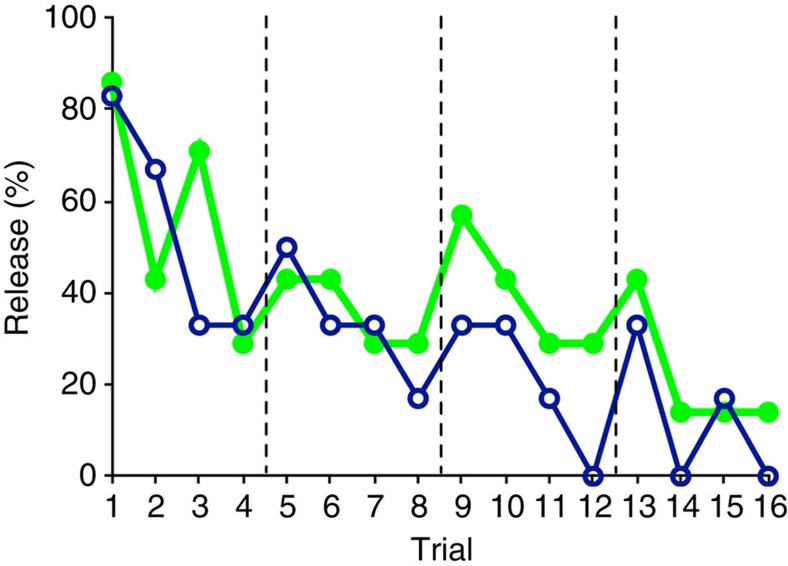
Results of experiment 1. Across four sessions, actors in both the GO group (blue, open circles) and NO-GO group (green, closed circles) showed a decline in the percentage of trials in which they released the peg. At the start of each session (dashed line) there is a spike in performance consistent with spontaneous recovery.

Actors were no more likely to release the peg to help the recipient in the GO group as they were to release it to block recipients' access to food in the NO-GO group (exact Mann-Whitney *U* test, *N*_GO_=6, *N*_NO-GO_=7, *U*=17.00, *P*=0.628; Fig. 2 and Supplementary Movies 1–3). Chimpanzees in both groups initially released the peg at high rates (83% in GO and 86% in NO-GO), but quickly declined to do so in subsequent trials (Spearman's rho: GO *r*_s_=−0.822, *N*=16, *P*<0.001; NO-GO *r*_s_=−0.703, *N*=16, *P*=0.002). Both groups showed a typical extinction curve consistent with lack of reinforcement, along with ‘spikes' at the start of each session (spontaneous recovery of stimulus attraction). In sum, chimpanzees in experiment 1 did not behave in a manner consistent with prosocially motivated helping: actors did not appear to intend the social outcomes that resulted from their actions and any social consequences did not appear to be intrinsically rewarding.

To determine what the actors understood of the effects of their choices in experiment 1, they were given a post-test knowledge probe (one session of four trials). Actors started in the peg room. Actors could access the food box (that is, the food room) via a raceway that connected both rooms. This control followed experiment 1, rather than preceded it, to test for any unintended learning effects on part of the actors (namely being personally rewarded for certain actions). During this knowledge probe, actors resumed peg releases, even in the NO-GO group for which this action was not personally beneficial (25% go, 61% NO-GO). While there was no significant difference between the two groups (exact Mann-Whitney *U* test, *N*_GO_=6, *N*_NO–GO_=7, *U*=8.00, *P*=0.073), there was a trend for more releases by the NO-GO group, for whom releasing was not even personally beneficial—actors in experiment 1 seemed to have failed to understand the affordances of the apparatuses by observing the effects on a conspecific.

### Experiment 2

**Figure 3:**
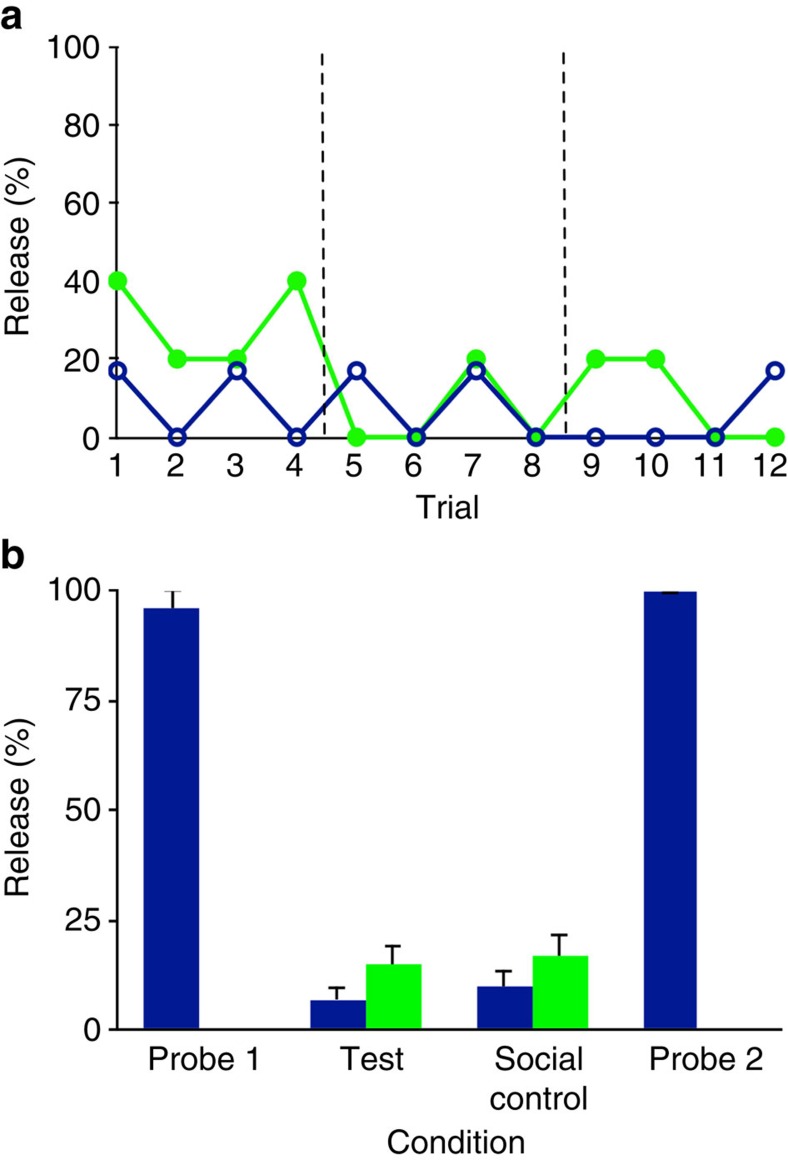
Results of experiment 2. (**a**) In the test conditions, chimpanzees in the GO group (blue) and NO-GO group (green) released the peg at low rates across the three sessions of four trials. (**b**) Release rates for actors in the GO group were at ceiling in the pre-test (probe 1) and post-test (probe 2) knowledge probes, whereas NO-GO group actors never released the peg (mean±s.e.m.). Release rates—for both groups—were very low in both the test and social control.

Actors were then given training sessions in which they individually learned about the respective food boxes' affordances. This is the approach taken by almost all previous studies on sharing and helping^20^,^21^,^22^,^23^,^24^,^26^,^27^,^28^,^29^,^30^,^31^,^34^,^35^,^36^. As in the previous knowledge probe, actors could move through the raceway from the peg room to the food room to access the food box. They were required to either release the peg before shaking the food box (GO group) or to inhibit releasing before shaking (NO-GO group). The criterion for success was to get food out of the food box in at least three consecutive trials. All but two actors (two chimpanzees in the NO-GO group) passed and were then given another knowledge probe. The training clearly improved understanding: actors in the GO group now released the peg 96% of the time and those in the NO-GO group never did so (0%; Fig. 3b). Following this, actors were again placed into the peg room and were again either paired with recipients in the food room (test trials—three sessions of four trials each) or with partners in an unconnected neighbouring room (testing for mere social presence effects: social control—three sessions of four trials each). Release rates in these test and control trials of experiment 2 were even lower than in experiment 1 (Fig. 3a). In the test trials, there was no difference in peg release between the GO and NO-GO group (exact Mann-Whitney *U* test, *N*_GO_=6, *N*_NO-GO_=5, *U*=13.5, *P*=0.792). Furthermore, there was no difference in peg release between the test and social control for these two groups (GO, Wilcoxon exact test: *z*=8, *N*=6, *P*=0.892; NO-GO group, Wilcoxon exact test: *z*=3, *N*=5, *P*=1; Fig. 3b). Following experiment 2, actors were given post-test knowledge probes, where they again demonstrated a clear understanding of the contingencies of the test: in the GO group actors always released the peg while the actors of the NO-GO group never did.

The results from these two experiments—in conjunction with the data from the knowledge probes—demonstrate that chimpanzees did not act to produce benefits for others in a helping context. Chimpanzees did not take into account the social consequences of their actions, even after having learned personally about the outcomes of their actions. Any benefits or harm to conspecifics that arose did so as incidental by-products of a personal interest in the stimulus, leading to the peg release actions. Actors showed an initial interest in the task (possibly even independent of social effects) which was quickly extinguished due to lack of reinforcement. Chimpanzees did not show any evidence for being motivated to influence outcomes that benefitted or harmed others.

## Discussion

The instrumental helping experiments presented here showed that chimpanzees will actively ‘help' conspecifics by performing a low-cost action that allows them access to food. However, they are just as likely to perform the same action when the effect is to prevent conspecifics from getting food. Chimpanzees, then, are no more prosocial than they are spiteful. Furthermore, regardless of outcomes for their partners, chimpanzees show a rapid decline in engaging with the task. Personal experience with the task only increases performance if the actors benefit personally from their actions. Chimpanzees do demonstrate an understanding of the consequences of their actions, but are indifferent to any effects on others.

These findings reconcile studies in which chimpanzees did not show signs of prosociality with those that did, and highlight the similarities between helping and sharing. In studies of active food sharing, chimpanzees failed to show a preference for prosocial outcomes when given a choice between outcomes that benefitted a conspecific from those that did not^19^,^20^,^21^,^22^,^30^. Some studies did report evidence for food sharing^24^,^25^,^31^. However, prosocial choices occurred at a fairly low rate, raising questions about how prosocial the subjects were. More critically, each of these studies is open to alternative explanations. In House *et al*.^24^, chimpanzees only showed weakly prosocial choices in a GO paradigm in which there was only one piece of food that could be delivered despite showing no prosocial preferences in other conditions. In a series of experiments by Claidiere *et al*.^31^ chimpanzees were faced with paired choices, one of which was mutually beneficial and one which was purely selfish; results were inconsistent and subjects failed knowledge controls. Perhaps the strongest positive evidence for sharing in an experiment comes from Horner *et al*.^25^ However, in this token exchange study, in which subjects preferred to exchange coloured tokens with an experimenter for wrapped food for both themselves and a partner, the results can be explained by a conditioned preference for the sound of food being unwrapped^45^; furthermore, there were no controls for task comprehension.

Instrumental helping studies in chimpanzees have shown more consistent evidence for prosociality, but here as well, alternative explanations have not been ruled out. First, the distinction between helping and sharing is not as clear-cut as has been suggested^37^,^38^,^39^. All instrumental helping studies with conspecific recipients involved delivering food or the means to get food^33^,^35^,^36^,^46^,^47^,^48^, blurring the distinction between sharing and helping. In the one direct test comparing prosociality rates when food or non-food items were delivered, there was no difference, showing that food delivery does not specifically impede prosocial behaviour^36^. The most highly cited evidence for helping in chimpanzee comes from experiments in which subjects hand objects back to human experimenters^32^,^33^. However, the chimpanzees had prior experience with handing objects back to their caregivers and were reinforced intermittently for this behaviour. A variable reinforcement schedule such as this produces persistent responding and it is not surprising that it generalized to a similar context in testing. Even though the behaviour of handing objects to humans was not intentionally trained for those studies, the prior learning history of the subjects has to be taken into consideration. When training is an explicit part of the experimental procedure, caution is needed in interpreting the results. In one study, chimpanzees that had been trained in symbolic use transferred food to their partners^49^. However, explicit training through standard shaping and training produced similar results in pigeons^50^, highlighting the importance of flexible behaviours in response to novel circumstances^35^. Other, more recent, helping studies^35^,^36^,^46^,^48^,^51^ can be explained by social tool use (giving a tool to a partner to get food for oneself), responding to solicitation (begging), or task persistence whenever food was visible, calling into question prosocial motivations.

One suggestion for the inconsistent evidence for prosociality in chimpanzees is that they are too competitive and that other species might be better models. Bonobos, which are as closely related to humans as are chimpanzees, have been suggested as having a more peaceful temperament^52^. Evidence for prosocial preferences has come from studies in which they open doors for conspecifics allowing them to co-feed^53^,^54^. However, when given prosocial preference tasks as used in chimpanzees, there has been no evidence for active food sharing in bonobos^19^,^55^. In the only test of instrumental helping in bonobos, there was no evidence of prosociality, with only the relatively solitary orangutans handing tools to distressed partners^47^. Other non-*Pan* ape species have shown no signs of sharing^19^,^30^,^56^, evidence in Old World primates has been mixed, with self-regarding preferences for social contact as possible explanations for apparent prosociality^29^,^30^,^57^,^58^,^59^. Among New World primates, capuchin monkeys have not consistently exhibited prosocial sharing^19^,^31^,^60^,^61^,^62^ although see^63^,^64^. They will ‘help' humans by passing objects to them in exchange for food^34^,^65^, although they do not transfer objects to help conspecifics get food^66^. The strongest evidence for prosociality comes from more distantly related New World monkeys that provide alloparental care for offspring, leading to the suggestion that cooperative breeding is a key driver for the evolution of primate prosociality^27^,^28^,^30^,^67^,^68^. However, these results have not been consistently replicated^26^,^69^ and various experimental details such as test order effects and partner presence cast some doubt on the evidence for sharing in these primates^70^ (though see ref. 71). As yet, there have been no tests of instrumental helping in callitrichines.

Almost all prior food delivery (sharing) and instrumental helping studies have involved training in which actors first learn through personal experiences the results of their actions. While helpful in demonstrating that the animals have learned the task contingencies, this can create an expectation for getting rewarded in the test context. While it is important to demonstrate task understanding, this could be done after testing, or between tests of naïve then trained subjects as done here (ABA design). By giving chimpanzees the opportunity to observe the consequences of their actions on others before giving them personal experience mitigates the food expectation while still demonstrating task comprehension. Chimpanzees are able to learn through others by observing them^72^, so there is no reason that they could not learn to help, or hinder, others solely on the basis of observed outcomes.

The difference between studies that find evidence for prosociality in chimpanzees and other primates and those that do not can be attributed to design features of the experiments. Experimental setups which contain stimuli that are sufficiently interesting for chimpanzees and other nonhuman primates initially elicit actions. Once a novel feature of the environment ceases to be engaging or of personal value, interest in it diminishes, without any consideration for how this affects other individuals. If prior studies of chimpanzee prosocial behaviour that simply presented a single choice (do something or do nothing) had been designed such that the outcome of an action was harmful rather than beneficial to a partner, then chimpanzees might have been considered to be spiteful. The key strength of the approach taken in our study is that the same action (release a peg) under the same stimuli (shaking food box) had two opposing effects. The GO/NO-GO approach is a more powerful method for teasing apart other-regarding motivations from self-interest, in contrast to all prior helping studies that only use a GO design. Stimulus enhancement is one important determinant of chimpanzees' apparent prosociality in experimental settings. By engaging with an interesting or novel feature of the environment, particularly when doing so has a history of providing rewards, chimpanzees can benefit others as incidental by-products. Social benefits can also arise as by-products if the subject is trying to gain social contact, play with the partner, signal dominance, respond to harassment and so on^73^. Self-interest, rather than concern for the welfare of others, could explain putative prosocial behaviour in chimpanzees.

It might have been the case that the stakes were too low in the experiments reported here and that in other contexts—for example, where peg release might free a conspecific from confinement—motivated aid might be elicited. But a general point highlighted by our studies is that prosocial motivations cannot be elucidated from prosocial actions without first controlling for intrinsic interest in experimental setup (for example, by using a GO/NO-GO method). Future studies on helping, sharing, comforting and informing will have to directly address the motivational substrate. Thus, even though chimpanzees have elsewhere shown to recognize something of the goals of others^74^, they appear to lack the motivation to see those goals realized. Studies on chimpanzees and other nonhuman animals can shed light on the origins of our own prosocial behaviour and its importance for large-scale nonkin cooperation.

## Methods

### Experiment 1

We tested 13 chimpanzees with three recipients (16 chimpanzees overall, see below). These animals were rescued from illegal wildlife trade and kept at the Ngamba Island Chimpanzee Sanctuary, a forested island in Uganda. The research was approved and reviewed by the local ethics committee of CSWCT (Chimpanzee Sanctuary and Wildlife Conservation Trust), the organization running the Chimpanzee Sanctuary in Uganda, as well as UWA (Ugandan Wildlife Authorities) and UNCST (Ugandan National Council for Science and Technology). The chimpanzees live freely on the island, and come in at night to the sleeping rooms where they were tested in the morning. Participation was voluntary and after testing the subjects re-joined the rest of the group. They were not food or water deprived. Subjects had previously participated in studies on cooperation and helping^33^,^36^,^75^,^76^,^77^,^78^,^79^, but the current setup involved a novel apparatus. For further details on the subjects, see Supplementary Table 1. These studies took place in the summer of 2011.

One subject (recipient) could interact with a Plexiglas box containing food (food box). A second subject (actor) could not get food from the box in the test conditions, but could release a wooden peg connected to the box. For one group of actors, releasing the peg freed the box, allowing the recipient to get food out by shaking it (GO group). For the other group of actors, doing so locked the previously functional (that is, food-delivering) box in place so that the food could no longer be extracted (NO-GO group). During tests, actor and recipient were in separate rooms of the sleeping area across a 2 m wide corridor used by experimenters and keepers: actors were in the room with peg access (peg room) and recipients were in the room with food box access (food room). Actors and recipients could not interact physically, but could see and hear each other, as well as the entire apparatus (that is, food box and attached peg). The peg and food rooms were bridged by an overhead ‘raceway' that was used during knowledge probes, but which was closed during test and control trials.

The food boxes for both groups of subjects (GO and NO-GO) were Plexiglas boxes fixed to the outside of the mesh of the recipient's room. The lower end of each food box was set 60 cm above the ground. Directly below each food box, a metal hopper channelled the food into the food room; the experimenter could also drop food down the hopper directly (Supplementary Fig. 1). Hopper and food box were both placed directly under the overhead raceway.

The food boxes could be directly accessed by the experimenters, but not by the subjects. Recipients could get food (shelled peanuts) only indirectly, namely by shaking the boxes using a chain attached to the bottom that led through a hole in the Plexiglas food box into the food room. The use of a chain and shaking boxes was designed to be noisy so as to attract the attention of the actors, as need for help may have to be signalled through instrumental actions or communication^36^. A series of trays inside the boxes limited the rate at which peanuts cascaded down to the opening at the bottom, necessitating repeated shaking—thus getting a few peanuts per shake.

The GO apparatus had a strong, inflexible cord running across the corridor to the peg attached to bars of the peg room. This static cord prevented the GO box from being shaken. However, once the peg was released, a small rubber cord attached to a metal angle allowed the food box to be shaken repeatedly, dispensing food in the food room. The NO-GO apparatus, on the other hand, had a strong rubber cord running across the corridor to the peg attached to the mesh of the peg room. This cord allowed the food box to be shaken repeatedly, causing food to come out—as long as the peg maintained tension on the rubber cord. Once the peg was released, the NO-GO food box fell flush to mesh of the food room, and could no longer be shaken (it lacked the small rubber cord of the GO apparatus). The GO apparatus was marked with blue tape and the NO-GO with green tape to facilitate coding. There is no reason to believe that these colours had any influence on the chimpanzees' behaviours.

There was no demonstration or familiarization phase for the actors prior to testing. This was done to avoid any unintended learning effects that could have come about from actors getting the food themselves. Actors had to learn about the consequences of their actions during the test trials, but they could easily see across the corridor, a distance that had been used in a prior helping study^36^.

During testing, actors and recipients were brought into their respective rooms (actors into the peg room; recipients into the food room). The doors and the raceway were closed so that none could access the others' room or other parts of the sleeping area during the test. The recipient that each actor started with was counterbalanced across actors. Following this, recipients were always exchanged every two trials, in a fixed order (after Asega came Baluku, followed by Mawa, then Asega, and so on). We kept this order across all studies.

At the start of each trial, actors were given distractor items (towels soaked in fruit juice) to prevent a ceiling effect for peg releases; also, they were given a 6 m long rope (that served as distractor to reduce random pulling behaviour); all of these were also done in prior helping studies^33^,^36^. Both actors and recipients were also distracted with single peanuts at the start of each trial to keep them in position while the peg was placed and the food box baited. The soaked towel and individual peanuts also served to maintain motivation, particularly for the actors who received no food rewards during the test.

When the actors were in position away from the apparatus, the experimenter showed them a handful of about 45 peanuts (a small handful) and then walked over to bait the food box by pouring the peanuts into the top shelf. He then placed the peg into the mesh of the actor's room and then signalled the start of the trial. All this while, the actor as well as the recipient were kept away from the apparatus by a human helper each who provided the actor with single peanuts. The helper aimed to ensure that the actor would observe the baiting of the food box, but would also ensure that the actor would not leave position prematurely. Before each trial, both helpers stopped providing single peanuts to actor and recipient, respectively, then the actor was given the juice-soaked towel, and then both helpers and the experimenter left the testing area: this was the start of a trial.

Each trial lasted 60 s regardless of outcome. After the 60 s, the experimenter blocked the apparatus so that no more food could be released by the recipients. For each actor there were four sessions with four trials in each session. To maintain the recipients' motivation we provided motivational trials: if they did not receive any food in three successive experimental trials, they were given a motivational trial with a 50% probability of getting food. If after this the recipients again did not receive food in another experimental trial, they again received a motivational trial. This continued until recipients received food in an experimental trial. To ensure that these motivational trials did not interfere with the actors' motivations and knowledge of the apparatus, actors were moved out of sight before motivational trials commenced.

After completing the 16 trials of the experimental phase, actors were given post-test knowledge probes to determine whether they learned about the effects of their actions on the apparatus through observation. They were given one session of four trials. Actors started in the peg room; there was no partner in the food room. Once in position, the door to the overhead raceway was opened, allowing them access to the food box by traversing over to the food room. The raceway access was closed after 60 s had passed and subjects were given an additional 60 s to gather the food from the apparatus. This protocol was followed whether subjects released the peg or not for both GO and NO-GO groups, that is, subjects would not necessarily get food, and they could remain in the peg room if they failed to cross. At the end of the trial (120 s in total), the experimenter locked the food box and the actor was moved back to the peg room unless already there (unless all four trails were finished, upon which the actor was let go to join the conspecifics in the outdoor area).

All trials were videotaped with Sony digital cameras. The primary measure of whether the peg was released or not by the actor was coded live. All reported tests are two-tailed. Twenty percent of the trials were coded for reliability by an assistant blind to the study's design and purpose. Reliability for whether the peg was released by the actor was excellent (Cohen's kappa 0.95).

### Experiment 2

Eleven chimpanzees from experiment 1 participated as actors in experiment 2; two from the NO-GO group failed to pass the training phase (Nkumwa, a female; and Kisembo, a male). The GO group consisted of six individuals (three males) and the NO-GO group had five (three males). The same three chimpanzees were used as recipients (Supplementary Table 1).

The setup and apparatus were the same as in experiment 1. One additional room was used for a social control condition; here, instead of being in the food room, the recipient was in a room adjacent to the peg room (that is, a room without access to either peg or food box). In this way, the recipient was still present (that is, the control was still social), but unable to interact with the setup.

The overall procedure was the same as in experiment 1. The key difference was the addition of familiarization/training trials in which the actors directly experienced the consequences of their actions on the apparatus during the training phase. Actors were also given knowledge probes before and after testing to ascertain that they had learned—and remembered—how to use the apparatus to their personal benefit. Furthermore, actors were also tested in a social control condition in which the recipient was present and visible, but in a third room in which they could not interact with the apparatus (see above). The number of sessions was reduced to three, rather than four, due to testing time constraints. Still, each session contained four trials (see Supplementary Table 2).

Actors were given the same test apparatus (GO or NO-GO) that they had interacted with in experiment 1. There were three steps in the training phase, all of which had to be passed before actors could advance to the testing phase. In step 1, actors were in the food room. The apparatus was in the same functional state for both the GO and NO-GO groups (configured so that shaking it would release food). The experimenter baited the apparatus with approximately 45 peanuts. One more peanut was dropped down the hopper so that no initial shaking would be required to get this one. The purpose of this was to attract the chimpanzees to the food box. Once the chimpanzee arrived at the food box, they were given a 60 s trial. They had up to ten trials in a session—with a maximum of two sessions—to reach criterion level of performance of shaking the food box at least five times in each of three successive trials. All subjects—with the exception of one male (Kisembo)—passed this criterion, and moved on to step 2.

In step 2, actors of both groups started in the food room as before and had 120 s to shake the food box to get food (it was no longer necessary to drop peanuts down a hopper to lure them to the food box). The experimenter then placed the peg into the mesh of the peg room. After the actor had shaken the food box (five shakes), the overhead raceway was opened, allowing actors the choice to leave the food room and go to the peg room to release the peg or not. Actors in the NO-GO group thus had to inhibit moving over and releasing the peg (since doing so would have locked the box into position and prevented the food from being accessible). However, the opening of the raceway allowed subjects from the GO group to make their food box functional: the GO group actors could move to the peg room, release the peg there and then go back to the food box to shake food out. As in step 1, there was a criterion level of performance. Actors had to be able to shake the food box in its functional state at least five times in three successive trials within a maximum of two sessions of ten trials; for the GO group, this meant releasing the peg prior to shaking, for the NO-GO group, this meant inhibiting releasing of the peg. All 12 remaining actors passed criterion and moved to step 3 of training.

In step 3, having learned to release the peg (GO group) and to inhibit releasing it (NO-GO group), actors now started the trial in the peg room. The trial began with the raceway to the recipient's room locked. Actors were distracted by slowly handing them peanuts (as in the test phase of experiment 1). As in the test of experiment 1 they were then shown a handful of peanuts, which was placed into the food box. The peg was then attached to the mesh of the peg room where the actor was and the raceway was then opened. GO actors had to release the peg before passing over the raceway to the food room. NO-GO actors had to instead inhibit releasing the peg, then to cross the raceway to be able to get food out of the food boxes. The same criterion as in step 2 applied, and again subjects had a maximum of two sessions of ten trials to reach criterion. Eleven actors reached criterion and thus passed the final step of training. Only one actor, Nkumwa in the NO-GO group, failed and was thus excluded from further testing.

The pre-test knowledge probe was the same as the post-test knowledge probe of experiment 1. Actors had access to the food box in the recipient's room via a raceway. There was a single session of four trials.

In the test, actors were given the same apparatus they had used in experiment 1 as well as the training phase of experiment 2. They were paired with recipients as in experiment 1.

In addition to the test condition with the recipient in the food room (as in experiment 1), a social control condition had the recipient sitting in the room adjacent to the peg room, fully visible to the actor. The actor was free to release the peg in the social control condition, but since the food room was empty, releasing the peg had no effect on the movement of the food box or consequences for a conspecific. The test and social control trials were presented in a blocked design counterbalanced across actors. That is, actors were given three sessions of each condition (either social control or test) before switching to the other condition. There were four trials in each session.

Following the test phase, actors were given four post-test knowledge probe trials, as described above for the pre-test knowledge probe, to determine whether they remembered their prior training.

Data coding and analyses were conducted as in experiment 1.

### Data availability

All summary data are included in the manuscript and supplement; requests for more detailed data collected for this study are available from the corresponding author on request.

## Additional information

**How to cite this article:** Tennie, C. *et al*. The nature of prosociality in chimpanzees. *Nat. Commun.* **7,** 13915 doi: 10.1038/ncomms13915 (2016).

**Publisher's note:** Springer Nature remains neutral with regard to jurisdictional claims in published maps and institutional affiliations.

## Supplementary Material

Supplementary Information Supplementary Figures and Supplementary Tables.

Supplementary Movie 1 In this example from the GO group (Experiment 2), the recipient, Baluku, (on the left) can shake the food box but cannot get food out unless a bolt holding it (on the right) is released by an actor in the room across the keeper's corridor. The actor (Okech) releases the bolt and as a consequence, Baluku is able to get the food by shaking the box.

Supplementary Movie 2 Shown here is an example of a nonrelease in the GO group (Experiment 2). Baluku, the recipient (left), is again paired with Okech (right) as the actor. Here, Okech does not release the bolt and Baluku does not get any food out.

Supplementary Movie 3 In the NO-GO group (Experiment 2), the recipient, Baluku, on the left is able to get food out of the food box by shaking it. The actor across the corridor (Indi) is attracted to the apparatus and releases the bolt. This action locks the food box in position, preventing the recipient from getting the food.
