# Supplementary material for: The nature of prosociality in chimpanzees
Source: Nat Commun. 2016 Dec 20;7:13915. doi: 10.1038/ncomms13915 (PMC5187495; doi:10.1038/ncomms13915)
Supplement: Supplementary Information — Supplementary Figures and Supplementary Tables. [file ncomms13915-s1.pdf]

Supplementary Information

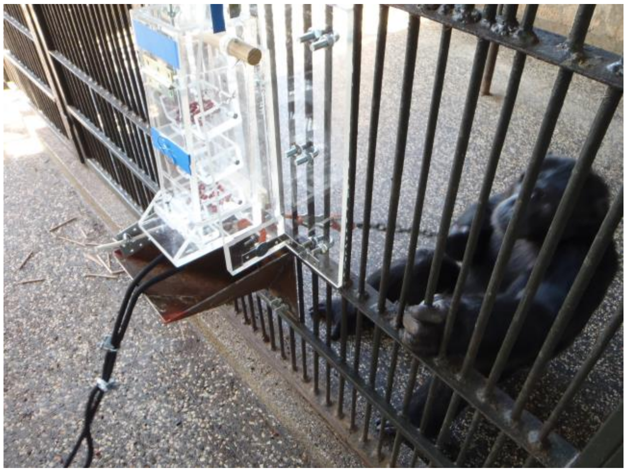

Supplementary Figure 1. Recipient shaking the food box in the go group.

Visible: the contained food trays that necessitate shaking to get the peanuts; the small white rubber cord that resets the apparatus (left of picture) - as well as the metal funnel below the food box.

Supplementary Table 1. Details of chimpanzees.

Name	Age (y)	Sex	Actors' Dominance	Treatment	Experiment
Recipients					
Asega (As)	13	M	n.a.	n.a.	1 & 2
Baluku (Ba)	13	M	n.a.	n.a.	1 & 2
Mawa (Ma)	15	M	n.a.	n.a.	1 & 2
Actors					
Ikuro (Ik)	16	F	none	go	1 & 2
Kalema (Ka)	15	M	As, Ba, Ma	go	1 & 2
Namukisa (Na)	12	F	none	go	1 & 2
Okech (Ok)	10	M	As, Ba	go	1 & 2
Umugenzi (Ug)	14	M	As, Ba	go	1 & 2
Yoyo (Yo)	12	F	none	go	1 & 2
Bili (Bi)	13	F	none	no-go	1 & 2
Bwambale (Bw)	12	M	none	no-go	1 & 2
Indi (In)	12	M	As, Ba	no-go	1 & 2
Kisembo (Ki)	12	M	none	no-go	1
Nani (Na)	10	F	none	no-go	1 & 2
Nkumwa (Nk)	15	F	none	no-go	1
Umutama (Ut)	15	M	As, Ba, Ma	no-go	1 & 2

Test subjects (recipients and actors) with details on their age, sex, dominance status relative to the recipients, treatments (go and no-go groups) and experiments they participated in (for the recipients, the treatments are not applicable).

Supplementary Table 2. Overview of the structure of Experiment 2.

Group	Session 1	Sessions 2-4	Sessions 5-7	Session 8
go	pre-test knowledge probe (probe 1)	test	social control	post-test knowledge probe (probe 2)
		social control	test	
no-go	pre-test knowledge probe (probe 1)	test	social control	post-test knowledge probe (probe 2)
		social control	test	

Note: Each session had four trials.
